# Allometric scaling of aerobic fitness outputs in school-aged pubertal girls

**DOI:** 10.1186/s12887-019-1462-2

**Published:** 2019-04-08

**Authors:** André O. Werneck, Jorge Conde, Manuel J. Coelho-e-Silva, Artur Pereira, Daniela C. Costa, Diogo Martinho, João P. Duarte, João Valente-dos-Santos, Rômulo A. Fernandes, Mariana B. Batista, David Ohara, Edilson S. Cyrino, Enio R. V. Ronque

**Affiliations:** 1 Study and Research Group in Physical Activity and Exercise (GEPAFE), State University of Londrina (UEL), Londrina, Paraná Brazil; 2 Study and Research Group in Metabolism, Nutrition, and Exercise (GEPEMENE), State University of Londrina (UEL), Londrina, Paraná Brazil; 3 Scientific Research Group Related to Physical Activity (GICRAF), Laboratory of InVestigation in Exercise (LIVE), Department of Physical Education, São Paulo State University, Presidente Prudente, São Paulo Brazil; 4 School of Health and Technology, Polytechnic Institute of Coimbra, Coimbra, Portugal; 5 CIDAF (uid/dtp/04213/2019), University of Coimbra, Coimbra, Portugal; 6 Faculty of Sports Sciences and Physical Education, University of Coimbra, Coimbra, Portugal; 7 Universidade de Coimbra, Estadio Universitario, Pavilhao III, 3040-156 Coimbra, Portugal; 8 Portuguese Foundation for Science and Technology (SFRF/BD/136193/2018), Lisbon, Portugal; 9 Portuguese Foundation for Science and Technology (SFRH/BD/121441/2016), Lisbon, Portugal; 10 Portuguese Foundation for Science and Technology (SFRH/BD/101083/2014), Lisbon, Portugal; 11 Portuguese Foundation for Science and Technology (SFRH/BPD/100470/2014), Lisbon, Portugal; 12 Institute for Biomedical Imaging and Life Sciences (IBILI), Faculty of Medicine, University of Coimbra, Coimbra, Portugal; 13 Faculty of Physical Education and Sport, Lusófona University of Humanities and Technologies, Lisbon, Portugal; 14 Federal University of Mato Grosso do Sul (UFMS), Pantanal Campus, Corumbá, Brazil

**Keywords:** Static allometry, Cardiorespiratory fitness, Body composition, Puberty growth spurt, Physical fitness

## Abstract

**Background:**

This study aimed to determine the allometric exponents for concurrent size descriptors (stature, body mass and fat-free mass) and also to examine the contribution of chronological age and pubertal status combined with above mentioned size descriptors to explain inter-individual variability in the peak of oxygen uptake (*V*O_2peak_) among girls during circumpubertal years.

**Methods:**

The final sample included 51 girls (10.7–13.5 years). *V*O_2peak_ was derived from an incremental progressive maximal protocol using a motorized treadmill. Anthropometry included body mass, stature and skinfolds. Measurements were performed by a single trained observer. Sexual maturation was assessed as self-reported stage of pubic hair (PH) development. Static allometric models were explored as an alternative to physiological output per unit of size descriptors. Allometry also considered chronological age and sexual maturation as dummy variable (PH2 vs. PH3 and PH3 vs. PH4).

**Results:**

Scaling coefficients for stature, body mass and fat-free mass were 1.463 (95%CI: 0.476 to 2.449), 0.516 (95%CI: 0.367 to 0.666) and 0.723 (95%CI: 0.494 to 0.951), respectively. The inclusion of sexual maturation increased explained variance for *V*O_2peak_ (55% for PH2 vs. PH3 and 47% for PH3 vs. PH4). Body mass was identified as the most prominent body size descriptor in the PH2 vs. PH3 while fat-free mass was the most relevant predictor combined with PH3 vs. PH4.

**Conclusions:**

Body mass and fat-free mass seemed to establish a non-linear relationship with *V*O_2peak_. Across puberty, inter-individual variability in *V*O_2peak_ is explained by sexual maturation combined with whole body during early puberty and by sexual maturation and fat-free mass during late puberty. Additional studies need to confirm ontogenetic allometric models during years of maximal growth.

## Background

Maximal oxygen uptake corresponds to the highest rate of oxygen consumption measured during incremental exercise test and reflects the cardiorespiratory fitness (CRF) of an individual during prolonged exercise. In addition, it is widely recognized as the best single measure of aerobic fitness [1]. All-cause mortality rates were studied in a large sample of men and women who were exposed to a preventive medical examination including physical fitness measured by a maximal treadmill exercise test [2] and conclusions demonstrated that high levels of physical fitness appeared to delay all-cause mortality primarily due to lowered rates of cardiovascular disease and cancer. Meantime, among children and adolescents, high levels of CRF were also believed to decrease the risk factors for cardiovascular diseases, such as obesity, high blood pressure, dyslipidemia, insulin resistance, among others [3].

Inter-individual variability in absolute peak oxygen uptake (*V*O_2peak_) is associated with functional characteristics and size of the lungs, heart and skeletal muscle [4]. Oxygen uptake is often expressed per unit of body size (body mass or fat-free mass) in the literature. Nevertheless, variation in body size is not easily summarized by a single anthropometric variable and although body mass is probably the most popular size descriptor, a recent study [5] considered alternative size descriptors, such as: whole body fat-free mass and lower-limb mass. The preceding observations were based on males spanning late childhood through adolescence who were involved in organized competitive soccer. Meantime, the standard ratio (mL·kg^− 1^·min^− 1^) has theoretical and mathematical limitations [6]. Scaling exponents are an alternative approach to accommodating inter-individual variability in body size regarding the interpretation of physiological variables as they provide size-free outputs [7].

Growth refers to the increase in the size of the body as a whole and of its parts. During the second decade of life, young people are expected to become taller and heavier showing an increase in lean and fat tissues, also, their organs increase in size. Heart volume and mass, for example, follow a growth pattern like that for body mass [8], while the lungs and their functions grow proportionally to stature. Adolescence is characterized by the growth spurt and sexual maturation. Maturation refers to progress towards the biologically mature state and should be viewed as a source of substantial inter-individual variability in almost all size descriptor. In the context of Physical Education, teachers should be careful in using cut-points. Many fitness batteries are associated with sex and age-specific tables to interpret performance values. This especially affects youngsters contrasting in biological maturation who need differential amount of time to keep working at improving.

Static allometric models can be useful to accommodate both size descriptors and maturity status or chronological age (CA) variable in the same model aiming to predict *V*O_2peak_ [9]. Therefore, this study aimed to estimate the allometric exponents for different size descriptors (stature, body mass and fat-free mass) as well as the contribution of CA and pubertal status combined with size descriptors to explain inter-individual variability in *V*O_2peak_ among girls during circumpubertal years.

## Methods

### Procedures

The current project was approved by the Research Ethics Committee for studies involving human participants of the State University of Londrina and University Medical Center of Northern Parana (CEP/UEL/202–07) and subsequently presented by the research leader to school boards and teachers. After obtaining institutional approval, a signed informed consent containing the objectives, protocols, and risks was obtained from parents or legal guardians and each participant. During the first visit, adolescents were informed that their participation was voluntary and that they could discontinue at any time. Procedures were conducted in accordance with the Declaration of Helsinki for human studies of the World Medical Association. Participants were recruited from schools in Londrina, State of Paraná, Brazil. After completing the data collection in the school, participants visited the laboratory of the *State University of Londrina.* The two data collections were performed within a 1-week period. *V*O_2peak_ was derived from a laboratory test using a motorized treadmill and a brief anthropometric battery was considered as part of the measurements during the final testing session. Indoor temperature in the laboratory was controlled to remain within the above-mentioned range (20–24 °C).

### Participants

The sample included 54 girls (10–13 years). Chronological age was calculated to the nearest 0.1 year from birth date minus testing date. The unique inclusion criteria were related to physical constraints that temporarily or permanently prevented the individual to participate in motor activities. Additionally, three adolescents were not included after checking for outliers.

### Sexual maturation

Pubic hair (PH) development was assessed using a self-assessment protocol based on drawings of the stages (1–5) as described by Tanner [10]. Participants were asked to compare themselves to the drawings and inform their perception regarding the similarity of their own PH development and stages summarized in the drawings. Due to the low prevalence of PH5, 4 adolescents were excluded from the main analysis.

### Anthropometry

Measurements were obtained by a single experienced observer using standardized procedures. Body mass and stature were measured to the nearest 0.1 kg and 0.1 cm using a scale (SECA 770, Hanover, MD, USA) and a stadiometer (Harpenden 98.603, Holtain Ltd., Croswell, UK), respectively. Skinfold thickness was assessed at the triceps and subscapular right side. Three measurements were obtained and the median value was retained for analysis. Technical errors of measurement were 1.02 mm and 0.72 mm for the triceps and subscapular, respectively, using a Lange caliper (Cambridge Scientific Instruments, Cambridge, MD) using standard technics [11]. Percentage of fat mass was estimated from skinfolds as independent variables [12] and fat mass and fat-free mass were subsequently calculated in kg.

### Peak oxygen uptake

Direct assessment of *V*O_2peak_ was conducted in a laboratory through open circuit spirometry with the performance of a progressive and maximum test on a treadmill ergometer. The test began with warm-up exercises for 3 min at a rate of 6 km∙h^− 1^ and 0% slope, after which the slope was increased to 1% and the speed increased by 1 km∙h^− 1^ every minute, maintaining the same slope, up to completion of the test. The protocol adopted was tested in a previous pilot study in which adolescents with the same characteristics and age reached maximum effort in a time interval between 8 and 12 min, which has been recommended to obtain aerobic power indicators in young people [13]. To measure *V*O_2_ during the test, a portable gas analyzer model K4 b2 (Cosmed, Rome, Italy) was used. The oxygen and carbon dioxide analyzers were calibrated before each test according to the manufacturer’s instructions. Respiratory parameters were recorded breath-by-breath, which in turn were averaged over a 15-s period. The criteria adopted for the completion of the test have been previously detailed [14] and were as follows: (a) subject’s voluntary exhaustion, with the request to finish the test; (b) reaching the maximum heart rate predicted for age (220 - age); (c) respiratory exchange ratio exceeding 1.1; (d) detection of a plateau in the VO_2_ curve, defined by an increase of less than 2 mL·kg^− 1^·min^− 1^ in the VO_2_ with change of stage in the test.

### Data analysis

Descriptive statistics (mean, standard deviation) were calculated for the total sample and the Kolmogorov-Smirnov test was used to assess normality. Appropriate logarithmic transformations were adopted to obtain normal distributions. Simple linear regression was adopted to estimate allometric coefficients of body size descriptors with *V*O_2peak_ as dependent variable. Equation 1 was used. Values of *a* and *k* were derived from linear regressions of the logarithmic regression transformations as illustrated by Eq. 2.

1 $$ y={a}^k\times \varepsilon $$

2 $$ \ln (y)=\ln (a)+k\times \ln (x)+\ln \left(\varepsilon \right) $$

Multiple linear regression was performed to examine the association between body size descriptors and chronological, as well as between body size descriptors and sexual maturation (dummy coded variables of PH: PH2 vs. PH3 and PH3 vs. PH4) – see Eqs. 3 and 4, respectively.

3 $$ \ln\ \left({\mathrm{VO}}_{2\mathrm{peak}}\right)={k}_1\times \ln \left(\mathrm{body}\ \mathrm{descriptor}\right)+a+\mathrm{b}\times \left(\mathrm{CA}\right) $$

4 $$ \ln\ \left({\mathrm{VO}}_{2\mathrm{peak}}\right)={k}_1\times \ln \left(\mathrm{body}\ \mathrm{descriptor}\right)+a+\mathrm{b}\times \mathrm{PH}\ \mathrm{stage}\ \left(\mathrm{dummy}\ \mathrm{coded}\ \mathrm{PH}\right) $$

All analysis were done using IBM SPSS 22.0 (SPSS, Inc., Chicago, IL). Significance level was set at 5% for all inferential statistics.

## Results

From the initial sample (*n* = 54), three girls were excluded due to missing data, thus the final sample was composed of 51 girls, from which, 21.6% were obese. Characteristics of the sample are described in Table 1. Aged ranged between 10.7 and 13.5 years. Dependent (*V*O_2peak_) and independent (stature, body mass and fat-free mass) variables assumed a normal distribution.

**Table 1:** Descriptive statistics for the total sample (*n* = 51) and test for normality

Variable	Unit	f	Range	Mean			Standard deviation	Komolgorov-Smirnov	
				Value	SEM	(95% CI)		Value	*p*
Pubic hair development									
Stage 2		16							
Stage 3		20							
Stage 4		11							
Stage 5		4							
Body mass index									
Normal weight		40							
Overweight/obese		11							
Chronological age	years		10.7–13.5	12.0	0.09	(11.8 to 12.2)	0.6	0.084	0.20
Stature	cm		147.9–151.7	149.8	0.96	(147.9 to 151.7)	6.8	0.067	0.20
Body mass	kg		27.6–75.3	43.5	1.51	(40.5 to 46.5)	10.8	0.118	0.07
Fat-free mass	kg		23.3–47.1	32.8	0.75	(31.3 to 34.3)	5.3	0.071	0.20
VO_2peak_	L∙min^−1^		1.06–2.55	1.80	0.04	(1.71 to 1.88)	0.30	0.112	0.20

*SEM* standard error of the mean, *95%CI* 95% confidence interval, *VO*_*2*_ oxygen uptake

Table 2 presents simple allometric models of *V*O_2peak_ using different size descriptors. The coefficients did not cross the linearity [body mass: 0.516 (95%CI: 0.367 to 0.666); fat-free mass: 0.723 (95%CI: 0.494 to 0.951)], with exception of stature [1.463 (95%CI: 0.476 to 2.449)]. The most explicative size descriptor was body mass (R^2^ = 0.486).

**Table 2:** Allometric modeling of VO_2peak_ using different body size descriptors (n = 51)

ln (VO_2peak_) = ln (*a*) + k × ln (Xi: size descriptor) + ln (ε)				Model summary	
Xi (size descriptor)	Constant	*k*-exponent		*R* ^2^	*Adjusted R* ^2^
		Value	(95% CI)		
Stature	−6.753	1.463	(0.476 to 2.449)	0.154	0.136
Body mass	−1.360	0.516	(0.367 to 0.666)	0.486	0.486
Fat-free mass	−1.939	0.723	(0.494 to 0.951)	0.452	0.441

*VO*_*2*_ oxygen uptake, *95% CI* 95% confidence interval

**Fig. 1:**
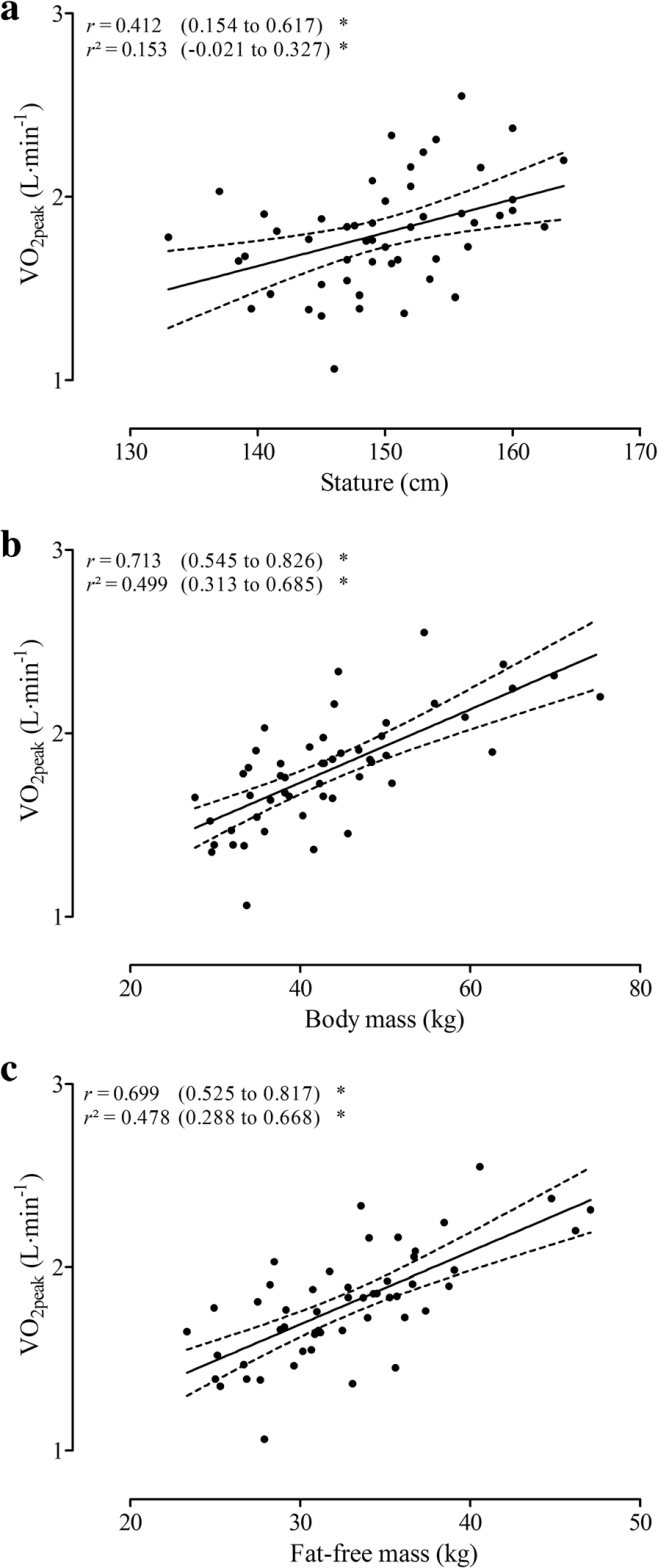
Linear regression of absolute *V*O_2peak_ directly measured and size descriptors [stature (panel **a**), body mass (panel **b**) and fat-free mass (panel **c**)]. * (*p* < 0.05)

**Fig. 2:**
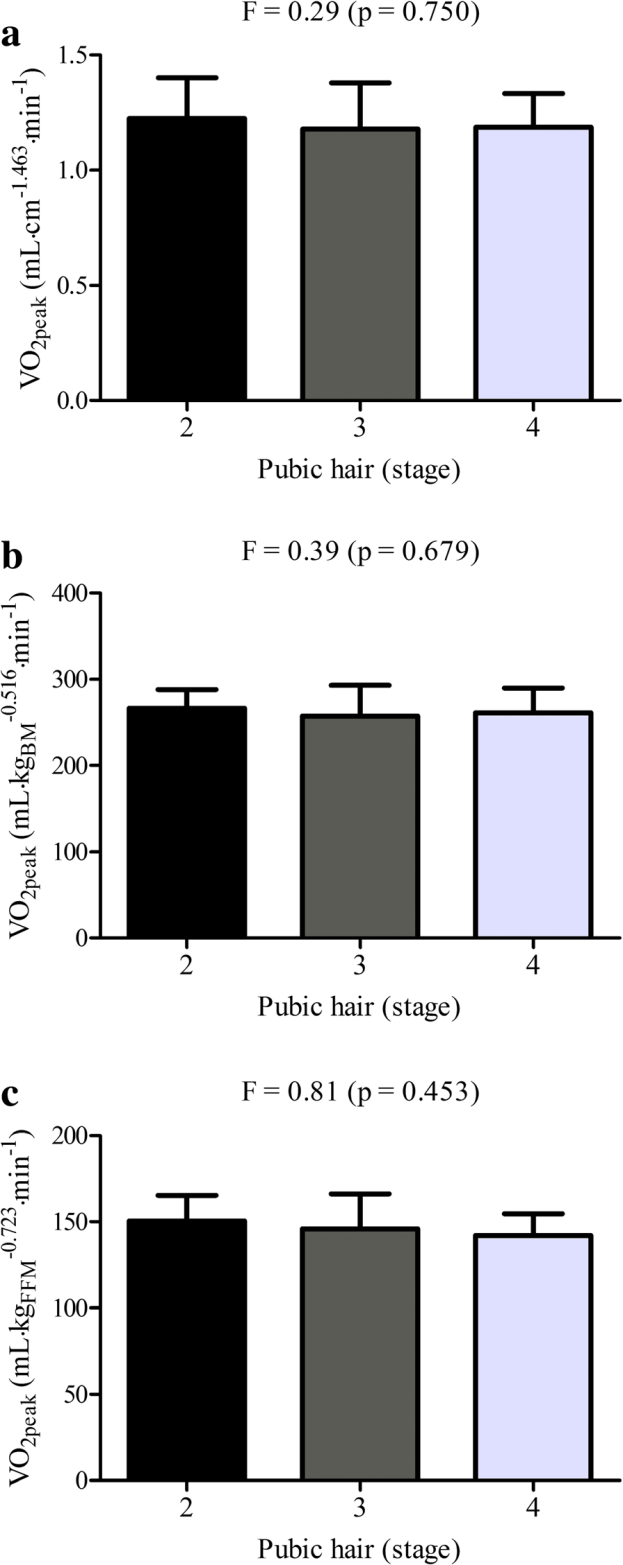
Mean values (± SD) of directly measured *V*O_2peak_ expressed by stature (panel **a**), body mass (panel **b**) and fat-free mass (panel **c**), considering the derived scaling coefficients, by stage of pubic hair (PH)

The association between crude size descriptors and *V*O_2peak_ is presented in Fig. 1. The most explicative size descriptor was body mass, with a r^2^ of 0.499. Moreover, scaled *V*O_2peak_ according to PH categories is presented on Fig. 2. PH groups presented no differences for all scaled *V*O_2peak_ (for stature, body mass and fat-free mass).

Multiplicative allometric models of VO_2peak_ combining different body size descriptors and CA are described in Table 3. In general, CA remained significant only in the model of fat-free mass and the explanation of the models did not changed substantially from simple allometric models, ranging between 16 and 48%.

**Table 3:** Multiplicative allometric modeling of the absolute VO_2peak_ combining body size descriptors with chronological age (n = 51)

ln (VO_2peak_) = k × ln (Xi: size descriptor) + *a* + b × (Z: chronological age) + ln (ε)					Model Summary		
*a*	Xi	Z	Exponent	(95%CI)	*R*	*R* ^*2*^	Adjusted *R*^*2*^
−7.144	Stature		1.676	(0.668 to 2.685)	0.443	0.196	0.163
		Chronological age	−0.058	(− 0.131 to 0.015)			
−1.010	Body mass		0.518	(0.369 to 0.667)	0.713	0.508	0.487
		Chronological age	−0.023	(− 0.078 to 0.033)			
−1.378	Fat-free mass		0.768	(0.544 to 0.992)	0.708	0.502	0.481
		Chronological age	−0.061	(−0.117 to − 0.005)			

*VO*_*2*_ oxygen uptake, *95%CI* 95% confidence interval

Table 4 presents the best multiplicative allometric models of *V*O_2peak_ combining body size descriptors and dummy stages of PH transitions (PH2 to PH3 and PH3 to PH4). The models included in the table were the models that best predicted *V*O_2peak_ in each transition of PH. Model of body mass was what best described VO2peak in the transition between PH2 and PH3, explaining 55% of *V*O_2peak_’s variation. Moreover, fat-free mass was the model that better described VO_2peak_ in the transition between PH3 and PH4, explaining 47% of the variation.

**Table 4:** Multiplicative allometric modeling of the absolute VO_2peak_ combining size descriptors and sexual maturation

ln (VO_2peak_) = k × ln (size descriptor) + *a* + b × PH stage (dummy coded) + ln (ε)					Model Summary		
*a*	Parameters						
	Size descriptor	PH stage	Exponent	(95%CI)	*R*	*R* ^*2*^	Adjusted *R*^*2*^
−1.505	Body mass		0.565	(0.392 to 0.737)	0.757	0.574	0.548
		PH2 vs PH3	−0.042	(−0.124 to 0.040)			
−2.814	Fat-free mass		0.978	(0.582 to 1.373)	0.710	0.504	0.468
		PH3 vs PH4	−0.054	(−0.165 to 0.057)			

*VO*_*2*_ oxygen uptake, *PH* pubic hair development, *95% CI* 95% confidence interval

Note: Only significant models were included

## Discussion

Aerobic fitness is an important component of physical fitness and is recognized as a protective factor for several negative outcomes as chronic diseases [15, 16] and mortality in adulthood [17]. It is often marked through *V*O_2peak_ that is consensually considered the best single indicator. During the adolescent years, CRF is associated with metabolic risk, independent of potential confounders [18, 19]. Variation in descriptors of body size and composition may be a consequence intervention for weight loss or training sessions aimed to increase muscle mass, but also of growth and biological maturation, especially during puberty growth spurt. By inference, caution is needed in the interpretation of absolute parameters of maximal oxygen uptake and also of traditional ratio. In this sense, power functions with allometric scaling exponents are proposed [9, 20, 21]. The current study used allometric scaling to evaluate the interrelationship among indicators of body size, composition, and parameters of aerobic fitness.

Growth refers to changes in body size and it has implications in proportionality, shape, and composition since different organs and tissues present distinct growth curves including the biological determinants of aerobic fitness such as heart and pulmonary sizes and functions [22–25]. Relationships among length, surface area and volume have implications for metabolism and thermoregulation [26]. Linear anthropometric descriptors such as stature are unidimensional. Areas including body surface area or muscle cross-sectional areas are bi-dimensional constructs (d = 2) and, finally, body volume is considered a tri-dimensional descriptor (d = 3). Meantime, considering time (minutes) as an uni-dimensional variable, absolute values of oxygen uptake measured in L·min^− 1^ corresponds to (d3-d1) and when oxygen uptake is divided by body mass in order to dissociate *V*O_2peak_ from body size (d3), isometric bodies stature corresponds to mass raised to 2/3 power function (assumption of geometric similarity). The current study obtained significant single allometric models that explained substantial percentages of inter-individual variability in *V*O_2peak_ (between 14 and 49%). As presented in Table 2, body mass was the best single predictor using allometric modeling. However, the scaling coefficient for body mass was 0.516 (95% CI: 0.367 to 0.666) which is below the exponent of 0.67 that provided support for geometric similarity [1, 26]. The mean values for body mass and stature in the current sample plotted at, or slightly above, age-specific 50th percentile of US reference data [27]. Surprisingly, the scaling coefficient for stature was near to linearity 1.463 (95% CI: 0.476 to 2.449) which was below the expected value suggested by the assumptions of geometric similarity [*V*O_2 (L·min-1)_ divided by stature _(cm)_ corresponds to 3–1 divided by 1]. Consequently, pubertal status was hypothesized as source of inter-individual variation among school girls. In multiplicative allometric equations (Table 3), the addition of CA to the models of size descriptors did not substantially change the prediction of *V*O_2peak_. Moreover, proportional static models with the inclusion of stage of PH development as dummy variables (PH2 vs. PH3 and PH3 vs. PH4) increased the explained variance (55% for PH2 vs. PH3 and 47% for PH3 vs. PH4) and body mass was kept as the most prominent body size descriptor in the PH2 vs. PH3 while fat-free mass was the most relevant predictor combined with PH3 vs. PH4.

The previous information confirms that simple ratio for reporting *V*O_2peak_ by size descriptors have limitations during puberty growth spurt [6]. The beginning of sexual maturation corresponds to increments in stature and body mass including non-isometric variation on intrinsic components affecting oxygen uptake such as heart size and pulmonary function [22–24]. Stages of puberty provide an indication of biological maturation. Secondary sex characteristics (breast and PH in girls, genital and PH in boys) are widely used, and at times misused, in studies of young athletes. Overt manifestation of breast, genital development and appearance of the first hair marks the transition into stage 2. During puberty growth spurt, peak height velocity occurs in stages 3, 4 and 5 of PH development. However, PH development represents the onset of adrenarche (increased secretion of hormones by the adrenal cortex) and not necessarily the onset of true pubertal development [10]. The percentage of females in each stage of PH development at attainment of peak height velocity and attainment of menarche were studied in 75 girls aged 8–15 years from Saskatchewan Pediatric Bone Mineral Accrual Study [28]. Menarche occurred later in PH development with the majority of the girls in PH4 (59.6%) and PH5 (31.9%) while peak height velocity was distributed in stage PH3 (42.5%) and PH4 (47.5%).

In the current study, fat-free mass emerged as the most relevant size descriptor with the progression of sexual maturation. Fat-free mass is the most metabolically active tissue [23, 29] and peak velocities of body mass and fat-free mass occurs in different moments and usually after peak height velocity that tend to occur at stage 3 and 4 [8]. Meantime, adolescence corresponds to a period of reduction in physical activity among girls with the increasing CA and biological maturation is viewed as mediating effect in lifestyle and intentions to exercise [30] which may also negatively contribute to aerobic fitness level.

A few limitations need to be recognized in the present study. The sample size was not large enough and did not equally cover the stages of sexual maturation. Body fat and fat-free mass were estimated from anthropometry (skinfold thickness) and currently, other methods are available (bioimpedance, air displacement plethysmography). Additionally, assessment of biological maturation was given by stages of sexual maturation which comprises several limitations [8]. Within the literature there is a substantial variability of average ages reported for adolescent attaining a stage of development for a secondary sex characteristic. For example, 11.56 years were reported for girls entering PH3 among 1494 girls from the cross-sectional Pécs Growth Study [31] which is substantially early compared to an average age of 12.84 years reported in third National Health and Nutrition Examination Survey [32]. Moreover, this last cited study also concluded that Non-Hispanic black girls had an earlier sexual development for PH and breast development either by median age at entry for a stage or for the mean age for a stage than Mexican American or non-Hispanic white girls. Studies devoted to ethnic variation of PH development in Brazil were not available. Skeletal age is recognized as the best indicator of biological maturation, although its application is costly and requires a small dose of radiation exposure and trained technicians [33]. Moreover, the prevalence of obesity in our sample was considerable 20%, what can influence the scaling coefficients and consequently, the results of the present study only can be inferred for non-athlete samples that present similar obesity rates. Finally, the cross-sectional design does not permit causality between size, maturation and maximal oxygen uptake.

The direct estimation of *V*O_2peak_ in school population and allometric approach should be recognized as the strength aspects of the present study and the data fits previous literature claiming for the analysis in young people [34]. The findings obtained in the current study have practical implications. Among untrained healthy school girls, stature seemed to present an almost linear relationship with maximal oxygen uptake. The three-dimensional size descriptors (body mass and fat-free mass) established a non-linear relationship with *V*O_2peak_ and across puberty inter-individual variability is explained by multiple combination of sexual maturation with body mass during mid-puberty and sexual maturation and fat-free mass during the transition for late-puberty to post-puberty.

## Conclusions

In conclusion, the traditional ratio by body mass should be used with caution to monitor the effects of Physical Education and other training or educational program regarding progresses of aerobic fitness throughout elementary school girls, given that the ratio by one unity of body mass can underestimate CRF of adolescent girls, especially with overweight and obese. Moreover, during the beginning of puberty (PH2-PH3), body mass was the best predictor of CRF, while fat-free mass was the best predictor of CRF during late puberty (PH3-PH4) and thus should be used in the ratio of *V*O_2peak_ with the appropriate allometric scaling coefficient for a better description of CRF.

## Abbreviations

CA: Chronological age
CRF: Cardiorespiratory fitness
PH: Pubic hair
*V*O_2peak_: Peak oxygen uptake
